# Comprehensive approach to preventing leishmaniasis in adults with nephropathy: a crucial imperative

**DOI:** 10.3205/dgkh000555

**Published:** 2025-06-06

**Authors:** Nader Aghakhani, Mehdi Azami, Mohammad Ali Mohaghegh

**Affiliations:** 1 Food and Beverages Safety Research Center, Urmia University of Medical Sciences, Urmia, Iran; 2 Department of Medical Parasitology and Microbiology, Hojjatieh Medical Diagnostic Laboratory, Hojjatieh Hospital, Isfahan, Iran; 3 Basir Laboratory Research and Development Center, Basir Medical Diagnostic Laboratory, Isfahan, Iran; 4 Department of Laboratory Sciences, School of Paramedical Sciences, Torbat Heydariyeh University of Medical Sciences, Torbat Heydariyeh, Iran; 5 Health Sciences Research Center, Torbat Heydariyeh University of Medical Sciences, Torbat Heydariyeh, Iran

## Letter to the editor

Leishmaniasis encompasses a range of diseases, from disfiguring cutaneous infections to potentially fatal visceral infections. It is caused by parasitic protozoa of the genus Leishmania, which are transmitted by infected sandflies [1]. Individuals with nephropathy, or kidney disease, are considered a vulnerable population at a higher risk of leishmaniasis due to their compromised immune system and impaired renal function. Although leishmaniasis can affect individuals of all age groups, adults with nephropathy are particularly susceptible due to their compromised immune systems [2]. The treatment of leishmaniasis in adults with nephropathy typically involves the use of antimony-containing compounds, such as sodium stibogluconate and meglumine antimoniate. Other medications that may be used include amphotericin B, miltefosine, pentamidine, and paromomycin. In cases of cutaneous leishmaniasis, plastic surgery may be necessary to repair the disfigurements on the face. However, it is always better to prioritize prevention over treatment.

Early screening and diagnosis of disease are necessary to prevent complications in adults with nephropathy. Regular screening, such as the soluble Leishmania antigen (SLA), is useful for monitoring patients who have undergone solid organ transplantation. It can aid in diagnosing of leishmaniasis in immunocompromised patients [3]. On the other hand, preventing sand-fly bites is essential for reducing the risk of leishmaniasis transmission. It is recommended to use insect repellents containing DEET (N,N-diethyl-meta-toluamide). But it must be considered, that DEET is not outright banned in most countries but is regulated for safety. For example, in Canada it is regulated with a proportion up to 30% for adults and 10% for children. In the European Union DEET is restricted up to 50%, in Japan up to <12% and in Australia and New Zealand up to <40% [4].

Alternatives to DEET are Picaridin. Its efficacy is comparable to DEET, but it is safe for all ages. IR3535 is effective and less irritating than DEET. Oil of lemon eucalyptu is natural, but less effective than DEET. Permethrin is only usable for clothing/gear, not for skin. Natural oils, e.g., lavender, peppermint are less effective than DEET and short-lasting [5], [6].

Additinally wear protective clothing, and avoid outdoor activities during peak sand-fly biting hours are important. Bed nets treated with insecticides can also provide an extra layer of protection. Clearing vegetation around households, applying insecticides to breeding sites, and implementing vector control programs have been proven effective in some endemic areas. Controlling sand-fly populations is crucial in preventing leishmaniasis transmission. Environmental management techniques, such as indoor residual spraying with insecticides, have shown efficacy in reducing sand-fly density and disease incidence [7]. 

Additionally, individuals with nephropathy who are planning to travel to regions where leishmaniasis is common should take specific precautions. They should be educated about the disease, instructed on proper personal protection measures, and advised to consult a healthcare professional before travelling for a risk assessment and appropriate prophylactic measures [8]. 

Managing nephropathy effectively is crucial in reducing the susceptibility of adult patients to leishmaniasis. Adequate maintenance of renal function, strict control of glycemic levels, and optimal management of blood pressure can help improve immune responses and overall health status.Raising health education and awareness among adults with nephropathy about the risks, symptoms, and preventive measures of leishmaniasis is of utmost importance. By providing educational materials, conducting seminars, and involving healthcare professionals, patients can be empowered to take appropriate precautions and seek early medical attention. Collaboration among nephrologists, infectious disease specialists, and primary care physicians is vital for providing comprehensive preventive care to adults with nephropathy. Regular communication, sharing of guidelines, and updating each other on emerging research findings help in implementing timely interventions and adopting a multidisciplinary approach [9]. 

In conclusion, preventing leishmaniasis in adults with nephropathy requires a multifaceted approach that involves screening, personal protection measures, environmental modifications, novel diagnostic tests, innovative approaches to vector control, and collaboration between healthcare provders. By implementing these preventive measures based on current evidence and expert recommendations, the burden of disease can be reduced, leading to better health outcomes for adult patients with nephropathy. Additionally, improving nutrition and supporting national control programs can help decrease the risk of infection. Treatment typically involves the use of antimony-containing compounds and other medicines. In cases of cutaneous leishmaniasis, plastic surgery may be needed.

## Notes

### Competing interests

The authors declare that they have no competing interests.

### Authors’ ORCIDs

- Azami M: https://orcid.org/0000-0003-2794-1508
- Aghakhani N: https://orcid.org/0000-0003-4026-2112
- Mohaghegh MA: https://orcid.org/0000-0001-8301-5256

### Funding

None. 
